# The ferroelectric photo ground state of SrTiO_3_: Cavity materials engineering

**DOI:** 10.1073/pnas.2105618118

**Published:** 2021-07-27

**Authors:** Simone Latini, Dongbin Shin, Shunsuke A. Sato, Christian Schäfer, Umberto De Giovannini, Hannes Hübener, Angel Rubio

**Affiliations:** ^a^Theory Department, Max Planck Institute for the Structure and Dynamics of Matter, Center for Free Electron Laser Science, 22761 Hamburg, Germany;; ^b^Center for Computational Sciences, University of Tsukuba, Tsukuba 305-8577, Japan;; ^c^Nano-Bio Spectroscopy Group, Departamento de Fisica de Materiales, Universidad del País Vasco, 20018 San Sebastián, Spain;; ^d^Center for Computational Quantum Physics, The Flatiron Institute, New York, NY 10010

**Keywords:** cavity materials engineering, quantum paraelectric to ferroelectric transition, strong light–matter hybrids, polaritons, SrTiO_3_ cavity phase diagram

## Abstract

Optical cavities confine light on a small region in space, which can result in a strong coupling of light with materials inside the cavity. This gives rise to new states where quantum fluctuations of light and matter can alter the properties of the material altogether. Here we demonstrate, based on first-principles calculations, that such light–matter coupling induces a change of the collective phase from quantum paraelectric to ferroelectric in the ${SrTiO}_{3}$ ground state, which has thus far only been achieved in out-of-equilibrium strongly excited conditions [X. Li et al., *Science* 364, 1079–1082 (2019) and T. F. Nova, A. S. Disa, M. Fechner, A. Cavalleri, *Science* 364, 1075–1079 (2019)]. This is a light–matter hybrid ground state which can only exist because of the coupling to the vacuum fluctuations of light, a *photo ground state*. The phase transition is accompanied by changes in the crystal structure, showing that fundamental ground state properties of materials can be controlled via strong light–matter coupling. Such a control of quantum states enables the tailoring of materials properties or even the design of novel materials purely by exposing them to confined light.

## Significance

Controlling collective phenomena in quantum materials is a promising route toward engineering material properties on demand. Strong THz lasers have been successful at inducing ferroelectricity in ${SrTiO}_{3}$. Here we demonstrate, from atomistic calculations, that cavity quantum vacuum fluctuations induce a change in the collective phase of ${SrTiO}_{3}$ in the strong light–matter coupling regime. Under these conditions, the ferroelectric phase is stabilized as the ground state, instead of the quantum paraelectric one. We conceptualize this light–matter hybrid state as a material photo ground state: Fundamental properties such as crystal structure, phonon frequencies, and the collective phase of a material are determined by the quantum light–matter coupling in equilibrium conditions. Cavity-coupling adds a new dimension to the phase diagram of ${SrTiO}_{3}$.

Engineering an out-of-equilibrium state of a material by means of strong light fields can drastically change its properties and even induce new phases altogether. This is considered a new paradigm of material design, especially when the collective behavior of particles in quantum materials can be controlled to provide novel functionalities (1, 2). Alternatively to the intense lasers necessary to reach such out-of-equilibrium states, one can achieve strong light–matter coupling by placing the material inside an optical cavity (3–11). A main advantage of this approach is that strong interaction can be achieved at equilibrium, opening up new possibilities for materials manipulation. Among the proposed effects are novel exciton insulator states (12), control of excitonic energy ordering (13), enhanced electron–phonon coupling (14), photon-mediated electron pairing (15–18), enhanced ferroelectricity (19), and multi-quasi-particles hybridization (20). One enticing possibility is, however, to change the ground state of a material and to create a new phase not through excited quasi-particles but truly as the equilibrium state.

Here we show that this can be achieved in the paraelectric ${SrTiO}_{3}$ as a photo-correlated ferroelectric ground state. This ground state, which we refer to as *photo ground state*, is the result of the strong coupling between matter and quantum vacuum fluctuations of light. While similar materials of the perovskite family undergo a para- to ferroelectric phase transition at low temperatures, ${SrTiO}_{3}$ remains paraelectric (21), because the nuclear quantum fluctuations prevent the emergence of a collective polarization that is characteristic of the ferroelectric phase (22, 23). Alterations to the material that overcome a relatively small activation energy, however, can induce ferroelectricity: for instance, through isotope substitution (24), strain (25, 26), and, most notably, nonlinear excitation of the lattice by strong and resonant terahertz laser pumping (27, 28). In the latter type of experiments, a transient broken symmetry of the structure as well as macroscopic polarization indicative of a transient ferroelectric phase have been observed.

By using atomistic calculations, we show that the off-resonant dressing of the lattice of ${SrTiO}_{3}$ with the vacuum fluctuations of the photons in a cavity can suppress the nuclear quantum fluctuations in a process that is analogous to the one of dynamical localization (29): As explained in *Results and Discussion*, the interaction with cavity photons effectively results in an enhancement of the effective mass of the ions, thus slowing them down and reducing the importance of their quantum fluctuations. We further demonstrate that the effect of cavity-induced localization extends to finite temperatures, even when thermal lattice fluctuations overcome the quantum ones. We thus introduce a revisited paraelectric to ferroelectric phase diagram, with the cavity coupling strength as a new dimension.

## Theory

**Fig. 1.**
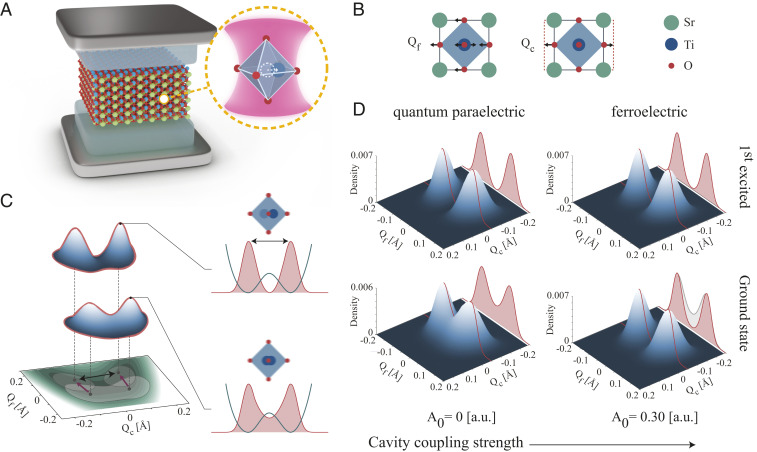
The emergence of ferroelectricity in ${SrTiO}_{3}$ in a dark optical cavity. (*A*) Cartoon of ${SrTiO}_{3}$ embedded in a dielectric medium inside a Fabry–Pérot cavity. The zoomed sketch shows the direction of the Ti–O displacive motion induced by the photons in the cavity. (*B*) Illustration of the main lattice motions involved in ferroelectricity: the ferroelectric mode and lattice vibration along the same direction, parameterized with $Q_{f}$ and $Q_{c}$, respectively. (*C*) Contour plot of the first-principles two-dimensional potential energy surface based on the Perdew–Burke–Ernzerhof functional and a schematic representation of the nuclear ground-state localization due to the coupling of the ferroelectric mode to the photons in the cavity. (*D*) First-principles density associated with the ground and first excited states outside the cavity ($A_{0}=0$ a.u.) and inside the cavity ($A_{0}=0.3$ a.u.) for a cavity frequency of 3 THz at zero temperature.

A microscopic theory that describes the structural and polarization properties of ${SrTiO}_{3}$ has to include nuclear quantum fluctuations, as confirmed by extensive literature (21–23). Ref. 30 develops a first-principles–based approach that includes the quantum mechanical nature of the ions and finds that the phases of ${SrTiO}_{3}$ can be properly described by specifically accounting for the quantum fluctuations of the nonlinearly coupled ferroelectric soft (FES) mode and lattice vibration; see sketch in Fig. 1*B*. In ${SrTiO}_{3}$, the quantum fluctuations of these two vibrational modes are strong enough to wash out the localization imposed by the double-well–shaped potential energy surface (Fig. 1*C*) and destroy the ferroelectric order stabilizing the so-called quantum paraelectric phase (24, 25). In this work, we introduce the paradigm of altering the localization and hence the macroscopic polarization properties of ${SrTiO}_{3}$ by coupling the FES mode to the confined quantized light modes of an optical cavity. The possible setup we consider in the following is a bulk film of tetragonal ${SrTiO}_{3}$ encapsulated in a transparent dielectric which is embedded in a Fabry–Pérot cavity as sketched in Fig. 1*A*. Here, we choose the ${SrTiO}_{3}$ crystal c axis to be parallel to the cavity mirrors and consider a single cavity photon mode with the smallest allowed momentum along the direction perpendicular to the mirrors. Since the displacement of the Ti–O atoms creates a dipole, the FES mode couples to the electric field of the confined photons. Despite the specific choice of the cavity geometry for the results presented in the following, the same ideas can be extended to other configurations or materials. The setup described above can be cast into an atomistic quantum electrodynamical (QED) Hamiltonian for the unit cell which reads $Ĥ=\omega_{c}{â}^{†}â+{\hat{p}}_{c}^{2}/2M_{c}+1/2M_{f}{\left[{\hat{p}}_{f}-A_{0}Z_{f}\left({â}^{†}+â\right)\right]}^{2}+V_{DFT}\left({\hat{Q}}_{c},{\hat{Q}}_{f}\right)$. This Hamiltonian describes the coupling of the zero-momentum FES mode, which is collective in nature, with a single cavity photon mode of frequency $\omega_{c}$ and with a coupling strength determined by $A_{0}Z_{f}/M_{f}$, where $A_{0}$ is the effective mode volume, $Z_{f}$ is the effective charge, $M_{f}$ is the effective mass of the FES mode, and DFT denotes density functional theory. Including the $A_{0}^{2}$ term guarantees the gauge invariance of the theory and the existence of a ground state (5, 31, 32). For details on all of the quantities, see *SI Appendix*. Our Hamiltonian builds upon the one reported in ref. 30 and properly describes the quantum paraelectric phase and the temperature dependence of the FES mode by including the fundamental phonon nonlinearities of ${SrTiO}_{3}$ via the potential $V_{DFT}$ calculated by DFT.

## Results and Discussion

The ground state and excited states of ${SrTiO}_{3}$ dressed with the quantized cavity photons can then be accessed via exact diagonalization of the QED Hamiltonian. The densities corresponding to the matter component of ground and first excited states for the material with and without coupling to the cavity photons are calculated by tracing out the photonic part and are reported in Fig. 1*D*. For ${SrTiO}_{3}$ outside the cavity, the ground state is different from the first excited state of the FES mode, which happens to be the first excited state of the QED Hamiltonian. It is a characteristic of a quantum paraelectric that, despite the double-well–shaped potential energy surface, the quantum fluctuations prevent the system from localizing in the wells. When the coupling to the cavity is turned on, the ground state and the first excited states become degenerate and indistinguishable, and present a clear node at $Q_{f}=0$. This is indicative of a ferroelectric phase: The system can choose to be in a linear combination of the two states where the Ti and O atoms have a finite positive or negative displacement. Any real system will then undergo spontaneous symmetry breaking and localize the FES mode in one of the two wells, leading to the formation of a macroscopic polarization, typical of the ferroelectric phase.

To summarize, by coupling the ${SrTiO}_{3}$ phonons to the cavity photons, we have realized the transition to a ferroelectric ground state. This phase transition can be explained in terms of the dynamical localization effects (29, 33), where the cavity dresses the masses of the lattice modes and thereby reduces their quantum fluctuations as depicted in Fig. 1*C*. Reducing the dimensionality of the system to the FES mode only and assuming a left localized and a right localized basis, the QED Hamiltonian can be simplified to $H=\omega_{c}a^{†}a+t\left({ĉ}_{R}^{†}{ĉ}_{L}+{ĉ}_{L}^{†}{ĉ}_{R}\right)-iA_{0}Z_{f}/M_{f}\left[\left({ĉ}_{R}^{†}{ĉ}_{L}-{ĉ}_{L}^{†}{ĉ}_{R}\right)\left(a+a^{†}\right)\right]$, where ${ĉ}_{L/M}^{†}$ and ${ĉ}_{L/M}$ are the creation and annihilation operators of the left and right states. If the hopping value t is chosen to be large enough to overcome the double-well potential barrier, the ground state of the Hamiltonian gives quantum paraelectricity. In the presence of the cavity photons, a simple effective Hamiltonian can be derived, as shown in *SI Appendix*, and in the high cavity frequency limit, it reads $H_{e\mathrm{ff}}=\left[t-{\left(A_{0}Z_{f}/M_{f}\omega_{c}\right)}^{2}t\right]\left({ĉ}_{R}^{†}{ĉ}_{L}+{ĉ}_{L}^{†}{ĉ}_{R}\right)-\frac{A_{0}^{2}Z_{f}^{2}}{M_{f}\omega_{c}}\left({ĉ}_{R}^{†}{ĉ}_{R}+{ĉ}_{L}^{†}{ĉ}_{L}\right)$. Hence the effect of the photon cavity is to localize the system by effectively reducing the hopping between the left and right states, $t\to t\left[1-{\left(A_{0}Z_{f}/M_{f}\omega_{c}\right)}^{2}\right]$. Physically, this can be interpreted as an enhancement of the effective FES mass $M_{f}$ induced by the vacuum fluctuations of light.

**Fig. 2.**
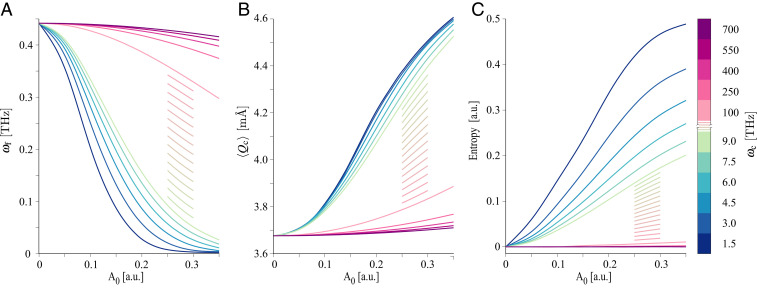
Dependence of the microscopic properties of ${SrTiO}_{3}$ on the cavity frequency $\omega_{c}$ and coupling strength $A_{0}$. (*A*) Generalized FES mode frequency, calculated as the difference in the energy of the nuclear first excited and ground states as a function of the cavity coupling strength for different cavity photon energies. (*B*) Expectation value of the mean displacement of the c lattice parameter as a function of the cavity coupling and photon energy. (*C*) The von Neumann entropy of the photonic subsystem as a function of cavity coupling and photon energy. This quantity indicates the degree of correlation going beyond the mean-field (Maxwell-like) light–matter coupling. The coupling strength is expressed in a.u., and all of the calculations are done at zero temperature.

In the following, we report on the dependence of the FES mode frequency, lattice displacements and subsystem entropy as function of the cavity coupling strength and the frequency of the cavity at zero temperature. In Fig. 2*A*, we show the FES mode frequency identified as the energy difference between the first excited state and ground state of the QED Hamiltonian. This difference is reduced with increasing cavity coupling, corresponding to a softening of the FES mode, which is indicative of the transition to the ferroelectric phase. However, we note that, when the system acquires a ferroelectric character, the FES mode is no longer a proper normal mode, because of the nonnegligible bilinear coupling with the lattice vibrations at the bottom of the wells. Therefore, we refer to this energy difference as the *generalized* FES mode frequency. The striking result is that ferroelectricity can be reached for a wide range of cavity photon energies; in other words, the cavity does not need to be resonant with the generalized FES mode energy (or any other phonon modes), and, indeed, the effect is larger off-resonance. This result is of technological relevance, since, in the recent experiments that report on laser-induced ferroelectricity, the laser had to be in resonance with the FES mode at 0.5 THz, which is a challenge for laser technology (27). Considering the expectation value of the squared ferroelectric displacement, shown in *SI Appendix*, we find that the largest localization of the FES mode is achieved at around $\omega_{c}=3$ THz. The expectation value of the lattice vibration displacement in Fig. 2*B* follows a similar trend, and, for increasing coupling to the cavity photons, we can observe a lattice expansion which has important consequences on the FES mode. Indeed, as shown in Fig. 1*C*, the double well for the expanded lattice deepens, and hence the localization of the FES mode is enhanced. This, together with the effect of dynamical localization, is the mechanism underlying the transition to ferroelectricity. The analysis of the photonic component of the ground state indicates that, even though the empty cavity is dark, the light–matter interaction creates a finite photon number. We therefore refer to the ground state inside the cavity as a photo ground state. As a measure of light–matter correlation, we evaluated the von Neumann entropy for the photonic subsystem; see *SI Appendix*. This quantity, reported in Fig. 2*C*, indicates whether the system can be represented as a simple tensor product of a matter and a photonic state. The entropy becomes nonzero and increases with increasing coupling which, in a ground state, can only be the result of vacuum fluctuations. While, in ref. 19, it was suggested that the ferroelectric phase of ${SrTiO}_{3}$ that is induced by external perturbation can be enhanced by the cavity–matter coupling, we demonstrated here that the ferroelectric phase can be reached as an unperturbed photo ground state, when the intrinsic phonon nonlinearities are accounted for. We expect intrinsic nonlinearities to be essential for inducing any type of equilibrium phase transition by a cavity, as they cannot be induced by the cavity–matter coupling itself.

The cavity coupling strengths and cavity frequencies considered here can be achieved by tuning the ${SrTiO}_{3}$ film thickness and the distance between the cavity mirrors. Because the light–matter coupling is proportional to $\sqrt{d_{⊥}/v}$, with $d_{⊥}$ and v as the thickness of ${SrTiO}_{3}$ and the unit cell volume, respectively, a coupling value up to $A_{0}=0.35$ a.u. can be achieved for a thickness up to $d_{⊥}\approx 20\;\mu$m, while a frequency in the range of 1 THz to 10 THz requires cavity lengths between $L_{⊥}∼300$ and $L_{⊥}\approx 30\;\mu$m. A more extensive discussion and the definition of the light–matter coupling are reported in *SI Appendix*.

**Fig. 3.**
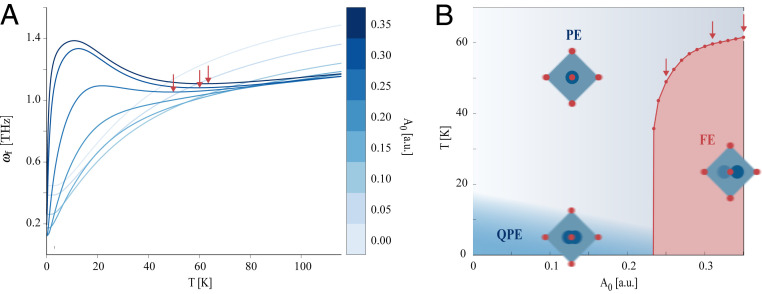
Revisited phase diagram of ${SrTiO}_{3}$ inside the optical cavity. (*A*) Effective microscopic ferroelectric frequency as a function of lattice temperature for different cavity coupling strengths. The minima of the curves, marked with red arrows, are used to identify the phase boundaries in *B*. (*B*) The microscopic phase diagram of ${SrTiO}_{3}$ inside the cavity. Three possible scenarios are opened up by the coupling to confined light. At low temperature, increasing the coupling with the cavity leads to a transition from a quantum paraelectric (QPE) to a ferroelectric (FE); at intermediate temperatures, increasing the coupling first allows thermal fluctuations to overcome the quantum ones and eventually reach a transition from paraelectric to ferroelectric; at high temperatures, since thermal fluctuations are much larger than the quantum ones, a direct transition from normal paraelectric (PE) to ferroelectric can be expected for increasing coupling to the cavity.

We now revisit the phase diagram of ${SrTiO}_{3}$ embedded in a quantum cavity, by adding the tunable coupling strength as a dimension to the diagram. Here, we suggest a microscopic approach to estimate a phase diagram by accounting for the effect of temperature via Kubo’s linear response theory on a thermal equilibrium state (see *SI Appendix* for details). We stress that such an approach neglects the phonon–phonon interactions and the temperature-dependent entropic effects arising from the excitation of higher momentum phonon modes which are not computationally feasible, but it allows us to keep our theory free of parameters. The response is calculated with respect to an external electric field coupled to the FES mode, representing a probe of ferroelectricity. The calculated response function for a cavity frequency $\omega_{c}=3$ THz is shown in *SI Appendix* and is characterized by a series of peaks whose intensity and width are changing with cavity coupling strength and temperature. In the case of no cavity coupling, the average of the frequency weighted by the response can be identified as the frequency of the FES mode which displays, as a function of temperature, a minimum resulting from a characteristic softening and a subsequent stiffening (34). This behavior, shown in Fig. 3*A*, is a hallmark of the phase transition from paraelectric to ferroelectric, and we therefore identify the transition temperature as the position of the minimum of the FES mode frequency. We stress that, despite the paraelectric to ferroelectric transition being a macroscopic collective phenomenon that involves the generation of a finite static polarization field, it leaves a signature on a microscopic dynamical quantity, the FES frequency, which can be used to estimate the transition. We then extend the frequency averaging procedure to finite cavity coupling and show the results in Fig. 3*A* where a distinctive trend can be observed for cavity couplings beyond a certain threshold. By tracing the evolution of the minimum that is characteristic of the paraelectric to ferroelectric phase transition, we are able to define a phase diagram which illustrates how the macroscopic phase of ${SrTiO}_{3}$ can be controlled by changing temperature and the cavity coupling strength. In the same phase diagram, we have also indicated the low-temperature quantum paraelectric phase with a gradient color, because the transition from paraelectric to quantum paraelectric is not an actual phase transition (35). The difference between a quantum paraelectric and a paraelectric can rather be understood in terms of whether thermal fluctuations prevail over the quantum ones. In this sense, we expect that the temperature at which the thermal fluctuations dominate decreases with increasing coupling strength, because the quantum fluctuations are suppressed by the cavity. A direct application of the phase diagram would be to prepare a paraelectric ${SrTiO}_{3}$ inside an optical cavity close to the phase boundary so that a laser resonant with a cavity photon can induce ferroelectricity. This approach has the advantage that the laser can be weak and not in resonance with the low frequency of the FES mode. This can be seen as enhanced ferroelectricity along the lines of what has been proposed in ref. 19.

## Conclusions

The concept of ferroelectric photo ground state illustrated by our atomistic calculations presents a paradigm for control of materials properties and opens avenues for materials engineering (11). The notable property here is that the vacuum fluctuations of the photon field dress the ground state and alter the crystal structure, lattice constant, and phonon frequency of the materials and even stabilize a macroscopic phase. Similar manipulations in other materials can be envisaged to yield control over quantum properties such as magnetic or even superconducting states.

## Supplementary Material

Supplementary File

## Data Availability

All study data are included in the article and *SI Appendix*.
