# Supplementary material for: The ferroelectric photo ground state of SrTiO3: Cavity materials engineering
Source: Proc Natl Acad Sci U S A. 2021 Jul 27;118(31):e2105618118. doi: 10.1073/pnas.2105618118 (PMC8346861; doi:10.1073/pnas.2105618118)
Supplement: Supplementary File [file pnas.2105618118.sapp.pdf]

Supplementary Information for

The Ferroelectric Photo-Groundstate of $SrTiO_3$: Cavity Materials Engineering

Simone Latini, Dongbin Shin, Shunsuke A. Sato, Christian Schäfer, Umberto De Giovannini, Hannes Hübener and Angel Rubio

E-mail: simone.latini@mpsd.mpg.de, angel.rubio@mpsd.mpg.de

This PDF file includes:

Supplementary text
Figs. S1 to S2
SI References

Supporting Information Text

1. QED Hamiltonian

In order to predict the properties of $SrTiO_3$ embedded in an optical cavity we introduce the following atomistic quantum electrodynamical (QED) Hamiltonian (1):

$$\hat{H} = \omega_c \hat{a}^\dagger \hat{a} + \frac{\hat{p}_c^2}{2M_c} + \frac{1}{2M_f}\left[\hat{p}_f - A_0 Z_f \left(\hat{a}^\dagger + \hat{a}\right)\right]^2 + V_{\mathrm{DFT}}(\hat{Q}_c, \hat{Q}_f), \qquad [1]$$

where ω_c is the frequency of the photons in the cavity which is set by the cavity length $L_\perp$, $a^\dagger$ and a are the corresponding creation and annihilation operators, $A = A_0(a^\dagger + a)$ is the cavity vector potential, $M_c = 111492$ a.u. and $M_f = 194059$ a.u. the effective masses of the lattice vibration (sum of the masses of the atoms in the unit cell) and the ferroelectric soft (FES) mode respectively, Z_f the Born effective charge of the FES mode and $V_{\mathrm{DFT}}(Q_c, Q_f) = \sum_{i=1}^{6} k_{f,i}\hat{Q}_f^{2i} + \sum_{j=2}^{5} k_{c,j}\hat{Q}_c^{j} + \sum_{i=1}^{6}\sum_{j=1}^{5} k_{fc,i,j}\hat{Q}_f^{2i}\hat{Q}_c^{j}$ the potential energy surface shown in Fig. 1(c) of the main text, which includes the intrinsic phonon non-linearities of $SrTiO_3$. The FES and lattice modes are parameterized in terms Q_f and Q_c respectively (see next section for more details on Q_f. The Born effective charges and the 2D potential energy surface expansion coefficients k are determined within DFT (density functional theory) using the Perdew-Burke-Ernzerhof (PBE) functional (2) as described in Ref. (3). Furthermore, we assume the Born-effective charge Z_f to be not affected by the light-matter coupling. We stress that in the Hamiltonian it is essential to take into account the diamagnetic as it guarantees the existence of a groundstate bound from below (4). It is important to note that in the Hamiltonian above we reduced the phononic degrees of freedom of $SrTiO_3$ to the FES mode and lattice vibration only and it is effectively describing a single unit cell. The unit cell Hamitlonian is effectively describing the collective Γ-phonon modes coupled with the dipole component of the electromagnetic fields. As shown in Ref. (3), this two modes are sufficient to correctly describes the physics of $SrTiO_3$ by demonstrating quantum paraelectricity and proving the correct behaviour of FES mode for an extended range of temperatures.

The strength of the cavity light-phonon coupling is determined by the Born effective charge and the photon mode amplitude A_0. For the Γ-phonon mode coupled to the dipole component of the electromagnetic field the mode amplitude is given by (5, 6):

$$A_0 = \sqrt{\frac{d_\perp}{2\pi c\ v}} \qquad [2]$$

with $d_\perp$ and v the thickness of the $SrTiO_3$ slab and the volume of the unit cell respectively, c the speed of light and we used the relation $L_\perp = \pi c/\omega_c$ where $L_\perp$ is the vertical dimension of the cavity. In order to obtain the expression above we took into account the difference in the thickness of the cavity and the material, i.e. $V_c = V_{\mathrm{SrTiO_3}} * L_\perp/d_\perp$. The scaling of the mode volume above with the thickness of the $SrTiO_3$ crystal provides a direct way to increase the coupling of the cavity increasing the amount of material.

2. Parameterization of Lattice Mode

In the atomistic Hamiltonian shown in the previous section we have conveniently decided to parameterize the FES mode with the distance between the Ti and O atoms along the c-axis, as this distance is ultimately involved in the definition of the unit cell dipole: $|\vec{D}| \equiv Z_f d_{\mathrm{Ti-O}}$. The particular choice however implies that the effective mass M_f and Born effective charge Z_f have to be rescaled accordingly as compared to the corresponding quantities for the FES phonon mode. This is because, even if of minor importance, the FES phonon mode involves the motion of all the other atoms in the unit cell. Since within density functional perturbation theory with the PBE functional (DFPT@PBE) (7) the FES mode has an imaginary frequency, the phonon mode is ill defined and therefore we evaluated such mode directly from the difference of the atomic position in the optimized paraeletric and ferroelectric geometry. Specifically, we defined the FES eigenvector as:

$$\vec{U}_I^{\mathrm{f}} = \frac{\vec{s}_I^{\ \mathrm{ferro}} - \vec{s}_I^{\ \mathrm{para}}}{\sum_J |\vec{s}_J^{\ \mathrm{ferro}} - \vec{s}_J^{\ \mathrm{para}}|}, \qquad [3]$$

where I is the index running over the atoms of the unit cell and $\vec{s}$ are the atomic basis vectors of the two different geometries. The eigevector above and Q_f are then related by:

$$Q_f = x_f\left(U_{\mathrm{Ti},z}^{\mathrm{f}} - U_{\mathrm{O},z}^{\mathrm{f}}\right) \qquad [4]$$

with x_f the actual FES mode parameter, i.e. the one that is independent of the specific atoms and to which the phonon effective masses and Born effective charges are standardly referred to. To calculate the effective mass and charge for the Q_f parameter, the following formula can be applied:

$$M_f = \sum_I M_I \left(\frac{U_{I,z}^{\mathrm{f}}}{U_{Ti,z}^{\mathrm{f}} - U_{O,z}^{\mathrm{f}}}\right)^2$$
$$Z_f = \sum_a Z_I \left(\frac{U_{I,z}^{\mathrm{f}}}{U_{Ti,z}^{\mathrm{f}} - U_{O,z}^{\mathrm{f}}}\right), \qquad [5]$$

where M_I and Z_I are the atomic mass and the Born effective charge for the atom I.

We set up our atomistic Hamiltonian for the $SrTiO_3$, outside the cavity, with the quantities defined above calculated using the PBE functional. The full diagonalization of the Hamiltonian is performed on a simple product basis set $|Q_\mathrm{f}\rangle \otimes |Q_\mathrm{c}\rangle \otimes |n\rangle$ which consists of a 50×25 real space grid for the phononic coordinates Q_f and Q_c and up to $n = 9$ Fock number states as a basis for the photons. We obtain a FES mode frequency of 0.44 THz, which reproduces well the experimental results (8–10).

3. Characterization of the Ferroelectric Photo-Groundstate

In the main text we characterized the photo-groundstate of $SrTiO_3$ in terms of the generalized FES mode frequency, mean displacement of the lattice vibration and Von Neumann entropy of the photonic sub-system. The latter quantity is commonly used to describe the amount of correlation between a given sub-system and all the others, which in our case is just the phononic system. The Von Neumann entropy is defined as:

$$\mathcal{S} = -\eta_i \sum_i \eta_i \log(\eta_i), \qquad [6]$$

where η_i are the eigenvalues of the density matrix of the chosen subsystem, which for the photons is defined as:

$$\hat{\rho}_\mathrm{ph} = \mathrm{Tr}_\mathrm{pn}\left[\hat{\rho}_\mathrm{full}\right], \qquad [7]$$

with Tr_pn meant as the trace over the phononic states. The resulting photonic entropy is the one shown in Fig. 2(c) of the main text.

To further characterize the photo-groundstate of $SrTiO_3$, we report the expectation value of the squared FES mode displacement, the purity and the expectation value of the photon number. These quantities are shown in Fig. S1 as a function of the coupling strength. We point out that the maximum of the mean squared displacement $\langle \hat{Q}_\mathrm{f}^2 \rangle$ is at $\omega_c = 3$ THz, which is off-resonant with the FES mode frequency.

The existence of an optimal for $\langle \hat{Q}_\mathrm{f}^2 \rangle$ is a consequence of the trade-off between the delocalization and the dipole matrix elements between the phononic states coupled by the cavity photons. Indeed the higher ω_c, the higher the phononic excited states that are coupled to the groundstate. In turns, this means that resulting delocalization gets larger but at the same time the dipole-matrix element becomes smaller.

Beside the Von Neumann entropy another way to characterize the correlation between light and matter is to evaluate the so-called purity (11). This is defined as follows:

$$\gamma = \mathrm{Tr}\left[\hat{\rho}_\mathrm{ph}^2\right]. \qquad [8]$$

A purity value that deviates from 1 means that the groundstate cannot be factorized in a simple tensor product of a phononic and photonic wavefunction, hence light and matter are correlated. In the context of correlated systems, another informative quantity is the so called inverse participation ratio (IPR). The IPR gives a measure of localization and it is defined as $\sigma = \sum_x \rho(x)^2$ with $\rho(x)$ the density of the system on a given space x. We calculated the IPR for the ferrolectric mode (we integrated out the Q_c dimension) with and without light-matter coupling for a cavity frequency of 3 THz: we found that the ferrolectric mode is more localized and the IPR ratio is $\sigma_{A_0=0.3}/\sigma_{A_0=0} = 0.017$.

Finally, the finite expectation value of the photon number operator $N_\mathrm{ph} = \langle \hat{a}^\dagger \hat{a} \rangle$ on the groundstate justifies the use of the term photo-groundstate. Indeed, even if the cavity is dark, there is a finite number of photons generated by the presence of $SrTiO_3$.

4. Dynamical Localization induced by Vacuum Fluctuations

In this section, we describe an alternative analytic simple approach to extend the theory of dynamical localization to the case of the quantized light field in a cavity (12, 13).

A. Effective Hamiltonian. The eigenvalue problem associated with the QED Hamiltonian in the first section can be rewritten in the following matrix form:

$$\begin{pmatrix} H_0 & H_1 & 0 & 0 & 0 & \dots \\ H_1^\dagger & H_0+\omega & \sqrt{2}H_1 & 0 & 0 & \dots \\ 0 & \sqrt{2}H_1^\dagger & H_0+2\omega & \sqrt{3}H_1 & 0 & \dots \\ \vdots & \ddots & \ddots & \ddots & \vdots & \end{pmatrix} \begin{pmatrix} u_0 \\ u_1 \\ u_2 \\ \vdots \end{pmatrix} = E \begin{pmatrix} u_0 \\ u_1 \\ u_2 \\ \vdots \end{pmatrix} \qquad [9]$$

where H_0 and H_1 are matrices in the matter basis (see below) and u_i are photon components of the eigenstate. The action on the n-th photon-sector can be written as

$$\sqrt{n}H_1^\dagger u_{n-1} + (H_0 + n\omega)u_n + \sqrt{n+1}H_1 u_{n+1} = E u_n, \qquad [10]$$

which can be proven by inspection. From this, one can recursively write the action of the full Hamiltonian matrix into a single photon sector in the form $H_\mathrm{eff}|u_0\rangle = E|u_0\rangle$ and thus get an approximation for the groundstate (or low lying eigenvalues). The effective Hamiltonian can be easily shown to be:

$$H_\mathrm{eff} = H_0 - H_1 \frac{1}{H_0 + \omega - E - 2H_1 \frac{1}{H_0 + 2\omega - E + \cdots} H_1^\dagger} H_1^\dagger. \qquad [11]$$

B. High frequency Approximation. How many photon sectors need to be included depends on the ratio between light-matter coupling and frequency. To make this clear we write $H_1 = A_0 P \tilde{H}_1$ and factorize:

$$H_{\text{eff}} = H_0 - \frac{(A_0 P)^2}{\omega} \tilde{H}_1 \frac{1}{1 + \frac{H_0 - E}{\omega} - 2\frac{(A_0 P)^2}{\omega} \tilde{H}_1 \frac{1}{H_0 + 2\omega - E} \tilde{H}_1^\dagger} \tilde{H}_1^\dagger \quad [12]$$

Only if $\omega \gg A_0 P$ the continued fractions can be neglected and the leading term reads

$$H_{\text{eff}} = H_0 - \frac{A_0^2 P^2}{\omega} \tilde{H}_1 \frac{1}{1 + \frac{H_0 - E}{\omega}} \tilde{H}_1^\dagger = H_0 - H_1 \left[H_0 + \omega - E\right]^{-1} H_1^\dagger, \quad [13]$$

as in the main text A_0 defines the photon mode volume and P is a c-number that sets the scale of the photon-phonon momentum matrix. Under this condition we can use the resolvent as a Neumann series and write

$$H_{\text{eff}} = H_0 - H_1 \sum_{n=0}^{\infty} \frac{(H_0 - E)^n}{\omega^{n+1}} H_1^\dagger. \quad [14]$$

The Neumann series converges for $\omega > \max(\{E_{0\lambda}\}) - E$, where $H_0 \psi_\lambda = E_{0\lambda} \psi_\lambda$, but Eq. (14) is only a valid approximation for the full Hamiltonian as long as the truncation at $n = 1$ is possible.

The series formulation of the effective Hamiltonian is useful because it yields to leading order in $1/\omega$:

$$H_{\text{eff}}^{(1)} \approx H_0 - \frac{H_1 H_1^\dagger}{\omega} \quad [15]$$

which can be readily solved. For all higher orders the effective Hamiltonain gives self-consistent eigenvalue equation that can only be solved iteratively. However, one can approximate this self-consistency by considering the linearization $E \to E_0$. To second order in $1/\omega$ the effective Hamiltonian for the ith eigenstate then reads

$$H_{\text{eff,i}}^{(2)} \approx H_0 - \frac{H_1 H_1^\dagger}{\omega} + \frac{H_1 H_0 H_1^\dagger}{\omega^2} - \frac{H_1 E_{0i} H_1^\dagger}{\omega^2} \quad [16]$$

which has to be solved separately for each eigenstate.

C. Localisation in $SrTiO_3$. To describe the photon induced localization we consider the system within a two-level approximation. We choose as the two levels, two Gaussians which are localized in the left and right well of the 1D FES mode energy potential respectively. In matrix form this translates to:

$$H = \begin{pmatrix} 0 & t \\ t & 0 \end{pmatrix} + A_0 P \begin{pmatrix} 0 & -i \\ i & 0 \end{pmatrix} (a + a^\dagger) + \omega a^\dagger a \quad [17]$$

so that $H_0 = t\sigma_x$ and $H_1 = A_0 P \sigma_y$. Here P is directly the L-R momentum matrix element. The high frequency approximation from the previous subsection then reads:

$$H_{\text{eff}} = \left(t - \frac{A_0^2 P^2 t}{\omega^2}\right) \sigma_x - \left(\frac{A_0^2 P^2}{\omega} + \frac{A_0^2 P^2 E_0}{\omega^2}\right) \mathbf{1}. \quad [18]$$

The second term is only shifting the eigenvalues, while the first one gives full localisation if $\frac{A_0^2 P^2}{\omega^2} = 1$. In that case the effective Hamiltonian has degenerate eigenvalues and similar to the full phonon-QED case one can make linear combinations of eigenvectors that give $(1, 0)$ and $(0, 1)$.

5. Temperature Dependent Response Function

In order to calculate the phase diagram that we presented in the main text, we need to include the effect of temperature in our theory. To do so we apply Kubo's formula for the linear response of a thermal state to a perturbation described by:

$$\hat{H}'(t) = -Z_{\text{f}} \hat{Q}_{\text{f}} E(t) \quad [19]$$

where Z_{f}, the FES mode effective charge, is assumed to be temperature independent. Applying Kubo's formula, the resulting polarizability takes the form:

$$\chi(\omega, T, A_0) = -\sum_{i,j} \rho_i(T, A_0) Z^* |D_{ij}(A_0)|^2 \times \left\{ \frac{1}{[\epsilon_j(A_0) - \epsilon_i(A_0)] - \omega - i\delta} + \frac{1}{[\epsilon_j(A_0) - \epsilon_i(A_0)] + \omega + i\delta} \right\}, \quad [20]$$

where the dipole matrix and the thermal density matrix are defined as $D_{ij}(A_0) = \langle \psi_i(A_0) | \hat{Q}_f | \psi_j(A_0) \rangle$ and $\rho_i(T, A_0) = e^{-[\epsilon_i(A_0) - \epsilon_0(A_0)]/k_B T} / \sum_j e^{-[\epsilon_j(A_0) - \epsilon_0(A_0)]/k_B T}$, respectively and $|\psi_i(A_0)\rangle$ are the eigenstates of the QED Hamiltonian for

different values of the cavity coupling. Such a response function for $SrTiO_3$ is shown in Fig. S2 for different temperatures and cavity coupling strengths. Note that the artificial broadening δ is kept constant with temperature however the increase in temperature introduces a finite population in the excited states which explains the appearance of further peaks.

We then define a characteristic temperature dependent FES mode frequency from such a response function as:

$$\omega(T, A_0) = \frac{\int d\omega\ \omega\ \mathrm{Im}[\chi(\omega, T A_0)]}{\int d\omega\ \mathrm{Im}[\chi(\omega, T, A_0)]}. \qquad [21]$$

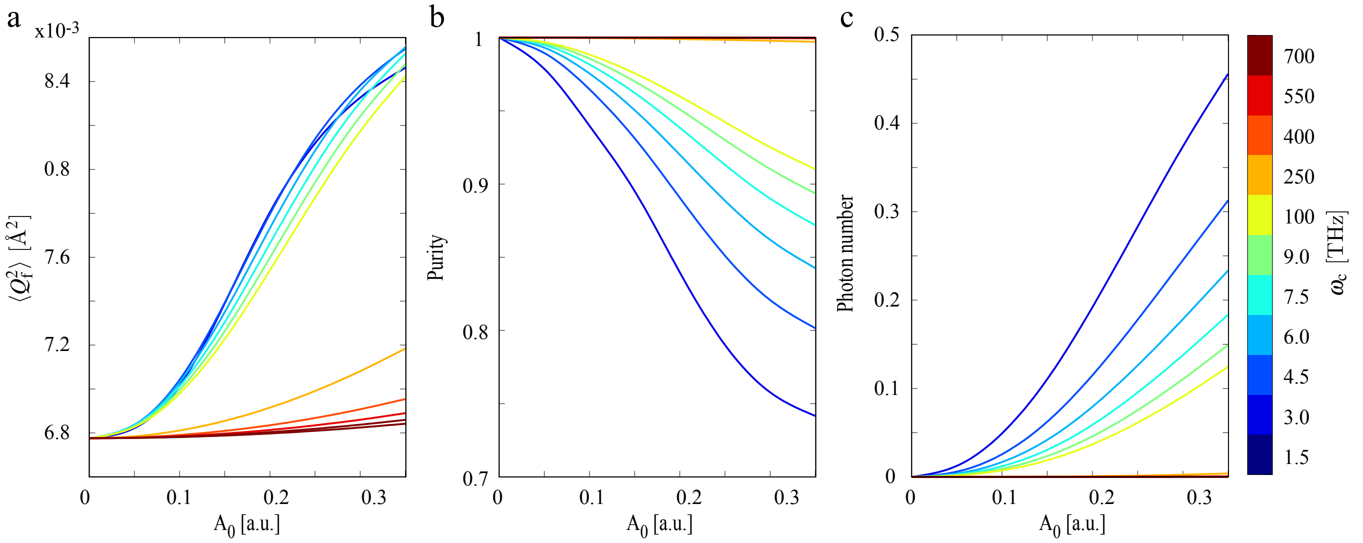

Fig. S1. **Dependence of the microscopic properties of $SrTiO_3$ on the cavity parameters.** (a) mean squared displacement of the generalized ferroelectric soft mode as a function of the cavity coupling and photon energy. Note that by the symmetry of the 2D potential energy surface the expectation value of the ferroelectric soft mode displacement has to be zero. (b) Purity of the photo-groundstate as a measure of light-matter correlation. (c) Expectation value of the number of photons.

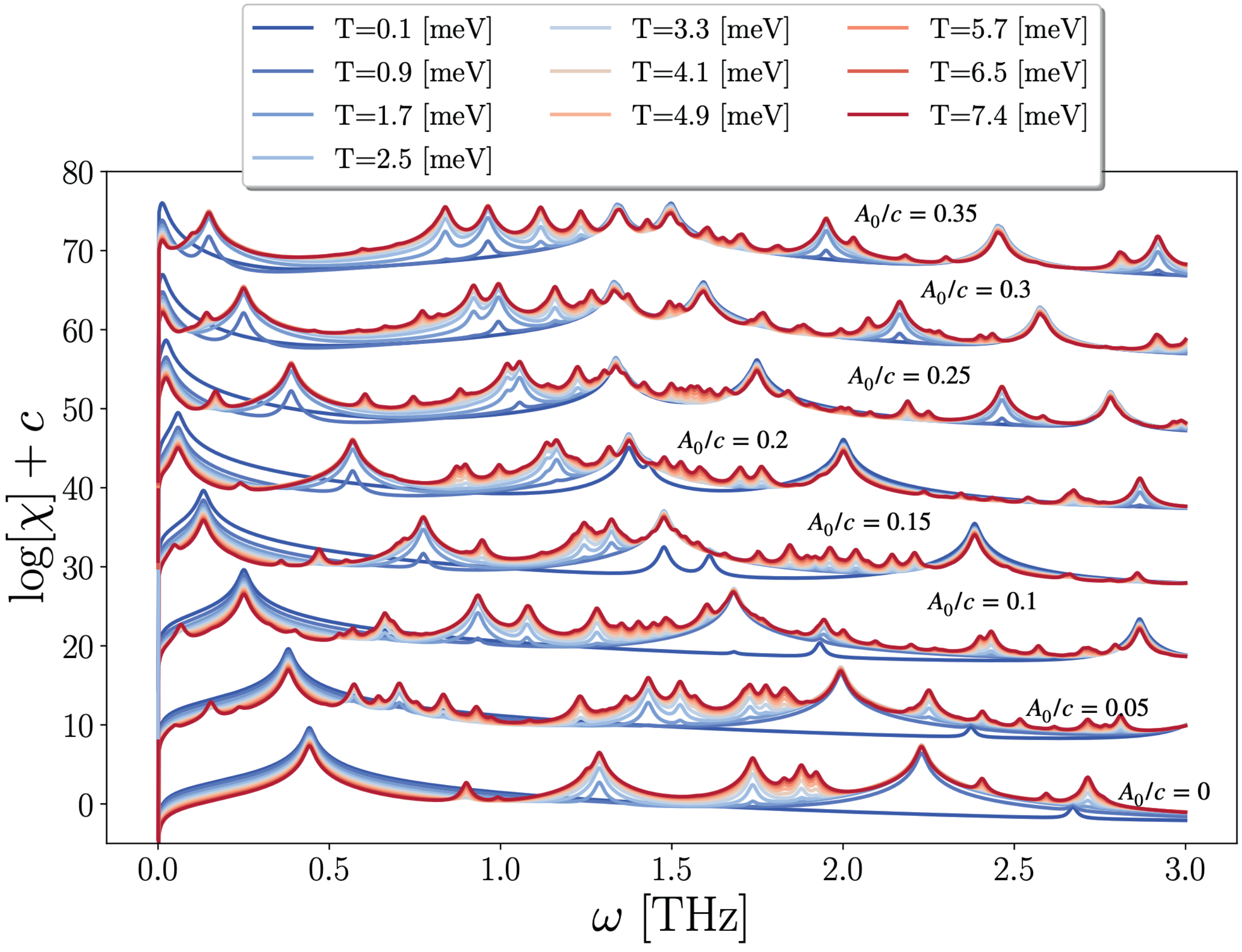

Fig. S2. Dependence of the imaginary part of the response function on temperature and cavity coupling strenght. The response to a probing electric field is calculated within Kubo's linear response theory. The values are shown in logarithmic scale and they are shifted by a constant for clarity.

References

1. M Ruggenthaler, N Tancogne-Dejean, J Flick, H Appel, A Rubio, From a quantum-electrodynamical light–matter description to novel spectroscopies. *Nat. Rev. Chem.* **2**, 1–16 (2018).
2. JP Perdew, K Burke, M Ernzerhof, Generalized gradient approximation made simple. *Phys. Rev. Lett.* **77**, 3865–3868 (1996).
3. D Shin, et al., The quantum paraelectric phase of $SrTiO_3$ from first principles. *arXiv preprint arXiv:2101.02291* (2021).
4. V Rokaj, DM Welakuh, M Ruggenthaler, A Rubio, Light–matter interaction in the long-wavelength limit: no ground-state without dipole self-energy. *J. Phys. B: At. Mol. Opt. Phys.* **51**, 034005 (2018).
5. Y Ashida, et al., Quantum electrodynamic control of matter: Cavity-enhanced ferroelectric phase transition. *Phys. Rev. X* **10**, 041027 (2020).
6. MA Sentef, M Ruggenthaler, A Rubio, Cavity quantum-electrodynamical polaritonically enhanced electron-phonon coupling and its influence on superconductivity. *Sci. Adv.* **4**, eaau6969 (2018).
7. X Gonze, C Lee, Dynamical matrices, Born effective charges, dielectric permittivity tensors, and interatomic force constants from density-functional perturbation theory. *Phys. Rev. B* **55**, 10355–10368 (1997).
8. G Shirane, Y Yamada, Lattice-dynamical study of the 110 K phase transition in $SrTiO_3$. *Phys. Rev.* **177**, 858 (1969).
9. A Yamanaka, et al., Evidence for competing orderings in strontium titanate from hyper-Raman scattering spectroscopy. *Eur. Lett.* **50**, 688–694 (2000).
10. H Vogt, Refined treatment of the model of linearly coupled anharmonic oscillators and its application to the temperature dependence of the zone-center soft-mode frequencies of $KTaO_3$ and $SrTiO_3$. *Phys. Rev. B* **51**, 8046–8059 (1995).
11. J Flick, M Ruggenthaler, H Appel, A Rubio, Kohn-Sham approach to quantum electrodynamical density-functional theory: Exact time-dependent effective potentials in real space. *Proc. Natl. Acad. Sci.* **112**, 15285–15290 (2015).
12. DH Dunlap, VM Kenkre, Dynamic localization of a charged particle moving under the influence of an electric field. *Phys. Rev. B* **34**, 3625–3633 (1986).
13. MA Sentef, J Li, F Künzel, M Eckstein, Quantum to classical crossover of Floquet engineering in correlated quantum systems. *Phys. Rev. Res.* **2**, 033033 (2020).
